# Correlation between thoracolumbar disc degeneration and anatomical spinopelvic parameters in supine position on MRI

**DOI:** 10.1371/journal.pone.0252385

**Published:** 2021-06-09

**Authors:** Sven S. Walter, Roberto Lorbeer, Gerald Hefferman, Christopher L. Schlett, Anette Peters, Susanne Rospleszcz, Konstantin Nikolaou, Fabian Bamberg, Mike Notohamiprodjo, Elke Maurer

**Affiliations:** 1 Department for Diagnostic and Interventional Radiology, Eberhard Karls University Tuebingen, University Hospital Tuebingen, Tübingen, Germany; 2 Department of Radiology, Ludwig-Maximilian-University Hospital Marchioninistraße, Munich, Germany; 3 Brigham and Women’s Hospital, Department of Radiology and Harvard Medical School, Boston, MA, United States of America; 4 Department of Diagnostic and Interventional Radiology, Medical Center ‐ University of Freiburg, Freiburg, Germany; 5 Institute of Epidemiology, Helmholtz Zentrum München, German Research Center forEnvironmental Health, Neuherberg, Germany; 6 Medical Faculty, Institute for Medical Information Processing, Biometry and Epidemiology, Ludwig-Maximilians-Universität München, Munich, Germany; 7 German Center for Diabetes Research (DZD), Partner site Neuherberg, Neuherberg, Germany; 8 Die Radiologie, Munich, Germany; 9 Department for Trauma and Reconstructive Surgery, BG Unfallklinik Tuebingen, Eberhard Karls University Tuebingen, Tuebingen, Germany; Johns Hopkins School of Medicine, UNITED STATES

## Abstract

**Objective:**

This study aims to investigate the correlation between spinopelvic parameters in supine position (pelvic incidence (PI), sacral slope (SS), pelvic tilt (PT), lumbar lordosis (LL)), disc degeneration and herniation of the thoracolumbar spine, as well as cardiovascular risk factors and back pain in a southern German cohort from the general population.

**Methods:**

This study is a cross-sectional, case–control study drawn from a prospective cohort of the “Cooperative Health Research in the Region of Augsburg/Kooperative Gesundheitsforschung in der Region Augsburg” study (KORA). In total, 374 participants (mean age 56.4 ± 9.2 years; 57.8% male) from the whole-body MRI cohort (FF4) were included. All participants underwent a standardized whole-body MRI on which disc degeneration of the thoracic and lumbar spine was evaluated using a sequence adapted Pfirrmann score. PI, PT, SS and LL were measured according to the description in the literature, using sagittal imaging. Furthermore, disc bulging and protrusion were assessed. Correlations were estimated by logistic regression models providing odds ratios.

**Results:**

Mean PI was 54.0° ± 11.1°, PT 13.0° ± 5.8°, SS 40.2° ± 8.8° and LL 36.2° ± 9.6°. SS was greater in men (p<0.05) and lumbar lordosis in women (p<0.001). PT increased by 0.09° per age-year with rising age. Age was not associated with PI, SS and LL. Neither BMI, hypertension, cholesterol, lipid levels, nor physical activity were associated with PI, PT, SS or LL. Diabetes mellitus negatively correlated with SS (β = -4.19; 95%CI -7.31–1.06, p<0.01). Smaller spinopelvic parameters (PI, SS and LL) where significantly (p<0.05) correlated with an increased frequency of disc bulging, as well as a local clustering in the lumbar, but not the thoracic spine.

**Conclusion:**

In conclusion, spinopelvic parameters, measured in supine position, are significantly correlated with disc bulging alone; there is no significant correlation between supine spinopelvic parameters and disc degeneration, back pain or cardiovascular risk factors.

## Introduction

There are several known risk factors for the development of intervertebral disc degeneration. Age [1–3], BMI [1,4–6], and level of physical activity [5,7] have been shown to correlate well with the risk of spinal degeneration and low back pain. Other cardiovascular risk factors, such as diabetes mellitus, hypertension, and serum lipid level, are controversially discussed as additional independent risk factors [8–11].

Spinal alignment is a critical element of postural stability, with pathologic alignment leading to degenerative changes, especially in patients with spinal fusion [12,13]. Recently, the geometric connection between sacrum and pelvis has been gaining attention as another potentially important parameter of postural control, as both the spinal profile and pelvic parameters characterize sagittal balance [14–16]. A distinction is made between morphological parameters, such as the pelvic incidence (PI), and functionally adjustable parameters, including sacral slope (SS), pelvic tilt (PT) and lumbar lordosis (LL) [16–18]. In contrast to the position-dependent parameters of the pelvis (PT and SS), the PI is individual for each person, correlates with age during growth, and is fixed at skeletal maturity [14,19–21].

To ensure an upright posture, the relation between the spinopelvic configuration and the physiologic kyphotic and lordotic bends of the spinal column is crucial, and is principally determined by sacral slope [19]. Boulay et al. and Yang et al. showed that the sacral slope, in turn, is characterized by the pelvic incidence, with small PI angles leading to a decrease in sacral slope, resulting in a flattening of the physiologic lumbar lordosis [22,23]. Loss of lumbar lordosis is considered to be a risk factor for degenerative alterations of the intervertebral discs [23]. However, few studies have directly investigated the association of the spinopelvic alignment and disc degeneration, with controversial results. X-rays are typically used to measure the spinal configuration, but are unable to characterize degenerative disc changes in detail. Thus, further characterization with MRI represents an important means of further evaluating spinopelvic configuration and intervertebral disc health [23].

Roussouly et al. demonstrated that there is no single physiologic unified sagittal balance, with four different anatomical configurations co-existing in the general population [19,24]. Importantly, the impact of these variations in sagittal balance on thoracolumbar intervertebral disc degeneration and low back pain remains poorly understood.

The purpose of this study was to investigate the correlation between spinopelvic parameters in supine position (PI, SS, PT and LL), disc degeneration and herniation of the thoracolumbar spine, as well as cardiovascular risk factors and back pain in a southern German cohort from the general population. This correlation may provide information about possible posture-related factors leading to chronic back pain or intervertebral disc degeneration, allowing for both prognostication and potential intervention if such a relationship exists.

## Material and methods

### Study design and sample

This population-based study used data collected during the “Cooperative Health Research in the Region of Augsburg” (KORA; Kooperative Gesundheitsforschung in der Region Augsburg) FF4-study, the second follow-up between 2013 and 2014 of the S4-study with baseline examinations during 1999–2001. The study design and protocol are described elsewhere [25].

Of the 2279 participants in the FF4-study, a subset of 400 subjects were included in the KORA-MRI study [25]. Besides the standardized interview and clinical examination of the FF4-study, all participants underwent a whole-body MRI.

Approval was given by the institutional review board of the Ludwig Maximilian’s University Munich (Germany). Each participant provided written consent.

### Covariates

Participants were classified as being diabetic if post-prandial serum glucose levels were ≥200 mg/dL after 2 hours after oral glucose tolerance test (OGTT) or fasting glucose levels were ≥126 mg/dL.

Hypertension was defined as either previously diagnosed with current hypertensive drug treatment or a recorded blood pressure of ≥130 mmHg systolic and ≥85 mmHg diastolic.

Potential excess weight of the participants was evaluated using the body mass index (BMI), which is defined as the subjects’ weight (in Kilogram) divided by height in meters squared (m^2^).

Additional measurements and reference values of serum high-density lipoproteins (HDL-c), low-density lipoproteins (LDL-c), total cholesterol, and triglycerides are described elsewhere [26].

Data regarding the degree of back pain was gathered twofold using a standardized questionnaire (during FF4). First, participants were asked if they suffered from back pain (Yes/No). Afterwards they had to further specify the intensity using a single-choice question with five graded answer options: A) none, B) little, C) moderate, D) strong and E) very strong.

### MR imaging protocol

Acquisitions of the whole-body MRI datasets were performed using a clinical 3-Tesla field strength scanner (Magnetom Skyra, Siemens Healthcare). A description of the technical background and exact image protocol is provided elsewhere [25]. In order to acquire anatomical parameters, a coronal dual-echo Dixon sequence and T2 SS-FSE (T2-HASTE) were acquired. The imaging parameters are shown in Table 1.

**Table 1:** Parameters of the acquired sequences.

	Weighting/Sequence type	Matrix	FoV (mm)	Voxel Size, In-plane (mm^2^)	Slice thickness (mm)	TR (ms)	TE (ms)	TI (ms)	Flip angle [°]
Dual-echo Dixon	VIBE	256 x 256	300 x 300	1.7 x 1.7	1.7	4.06	1.26; 2.49	N/A	9
HASTE	T2	320 x 200	296 x 380	1.2 x 1.2	5	1000	91	N/A	131

HASTE: Half Fourier acquisition single shot turbo spin echo; VIBE: Volume interpolated breathhold examination; T2: T2 weighted.

Before the examination, all participants were positioned according to a standardized protocol; supine position in the center of the table with slightly bend, but parallel legs, as well as arms parallel to the body. If participants were still misaligned in the pelvic region on the imaging datasets a 3D reconstruction was used to align the axis on a perpendicular line through both centers of the femoral head.

### Spinopelvic parameters

Spinopelvic balance comprises the morphologically existing parameter pelvic incidence, and the functionally adjustable parameters sacral slope, pelvic tilt and lumbar lordosis, as described by During and Duval-Beaupère [17,27].

#### Pelvic incidence

**Fig 1:**
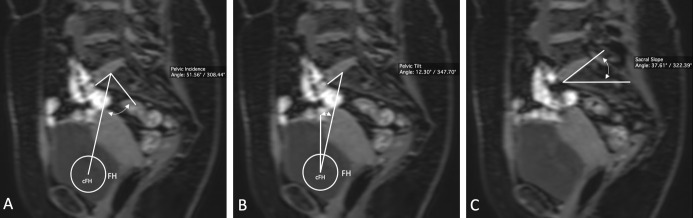
Measurement of pelvic incidence (A), pelvic tilt (b), sacral slope (C) and lumbar lordosis (D). Bent double arrow: Measured angle; FH: Femoral head; cFH: Center of the femoral head.

Pelvic incidence (PI) was defined as the subtended angle between the perpendicular line running through the center of the upper plate of the first sacral vertebra and the line linking this point to the femoral head axis (Fig 1A) [18,21]. PI can be additionally determined by the sum of the sacral slope and pelvic tilt (PI = SS + PT) due to the geometric relationships described below [18]. The accepted physiologic value of PI is 53.1 ± 9.0°, with gender exerting no effect on the measured variable [22].

#### Pelvic tilt

Pelvic tilt (PT) is defined as the angle between a line from the center of the sacral plateau to the center of the femoral head and the coronal plane (Fig 1B). In the upright position, the mean PT angle is 13 ± 6° [28].

#### Sacral slope

Sacral slope (SS) is defined as the angle of the sacral plate to the horizontal plane (Fig 1C) [28], and defines the foundation of the spinal column [14,18,22]. In the upright position, the mean SS is 41 ± 8° [29].

#### Lumbar lordosis

**Fig 2:**
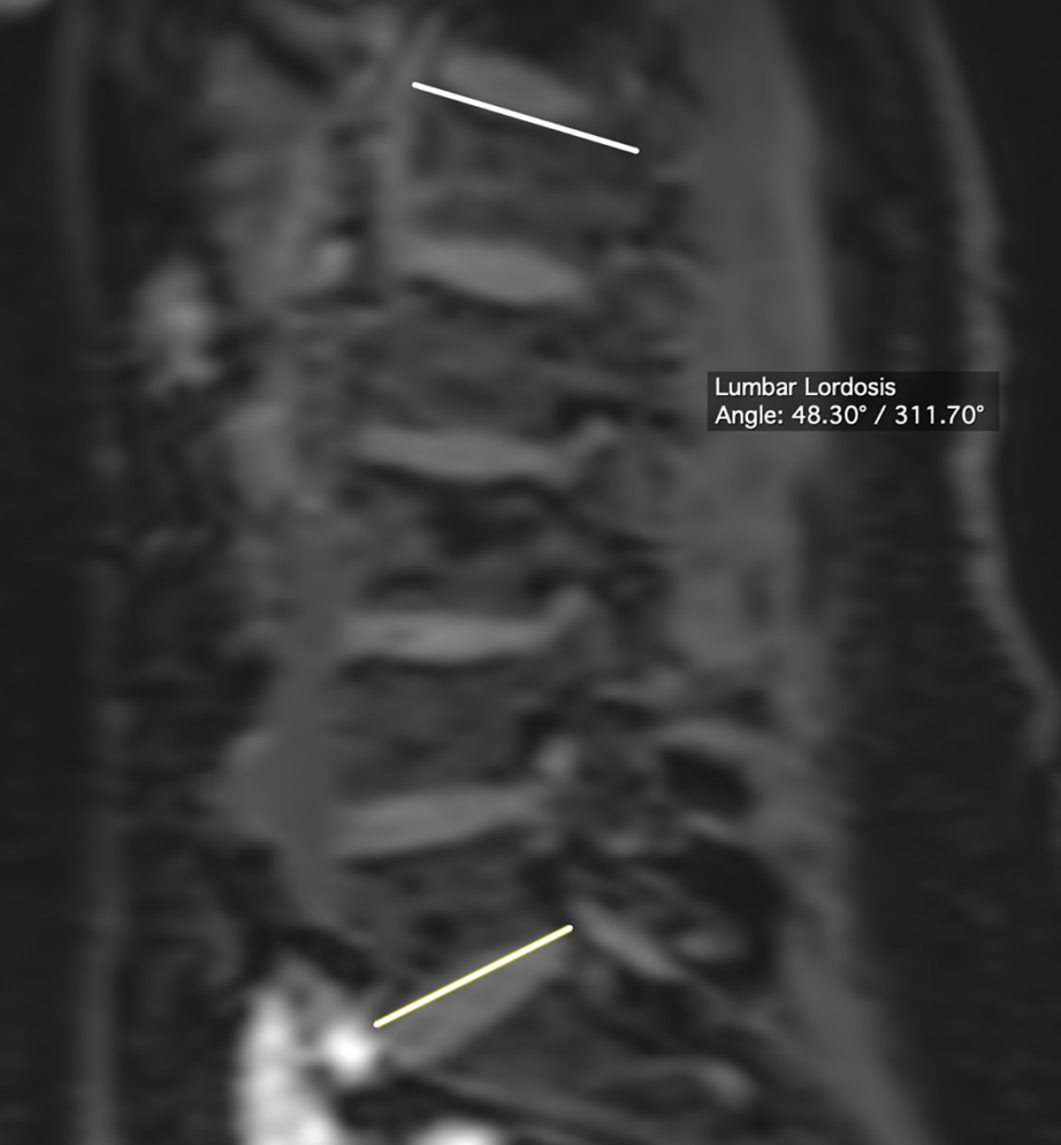
Measurement of lumbar lordosis.

Lumbar lordosis is defined as the angle between the superior plate of the first lumbar vertebra (L1) and the inferior plate of the last lumbar vertebra (L5) (Fig 2) [14,23]. In the upright position, the mean LL is 44 ± 11° [29].

### Disc degeneration and disc bulging/protrusion

**Fig 3:**
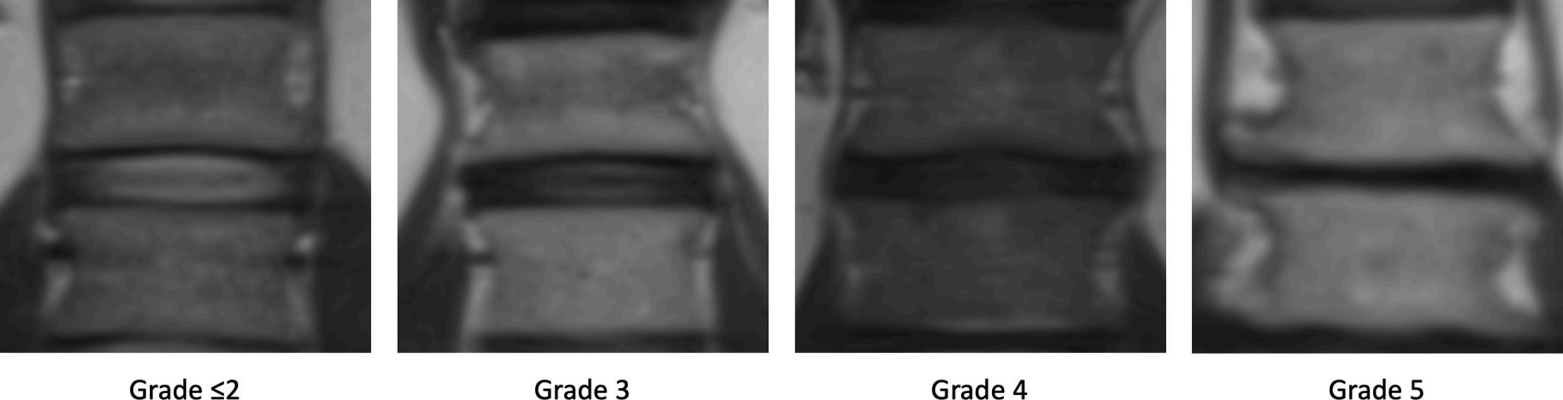
Adapted Pfirrmann grading for intervertebral disc degeneration L1/2 on a T2-HASTE sequence.

Degenerative changes of each intervertebral segment from thoracic vertebra 1 to lumbar vertebra 5 were assessed on a T2-HASTE sequence (Table 1) by a sequence adapted Pfirrmann score [30]. Briefly, the score is divided into 5 grades, whereby the increasing severity of disc degeneration shows a decrease in intervertebral height, distinction of the disc, and loss of symmetry (Fig 3) [7,30,31]. From this, the mean Pfirrmann score was calculated (“mean adapted Pfirrmann Score”). To assess the number of disc degenerations per participant, another dichotomous variable (“summed adapted Pfirrmann score” with “0” = healthy discs and “1” = disc degeneration) was generated, with grade 1 and 2 designated as healthy and grade 3 to 5 termed as degenerative discs. Thus, for 17 intervertebral spaces (overall: 17 segments; thoracic: 12 segments and lumbar 5 segments) of the thoracolumbar spine, values between 0 = no segment affected and 17 = 17 segments affected could be generated.

**Fig 4:**
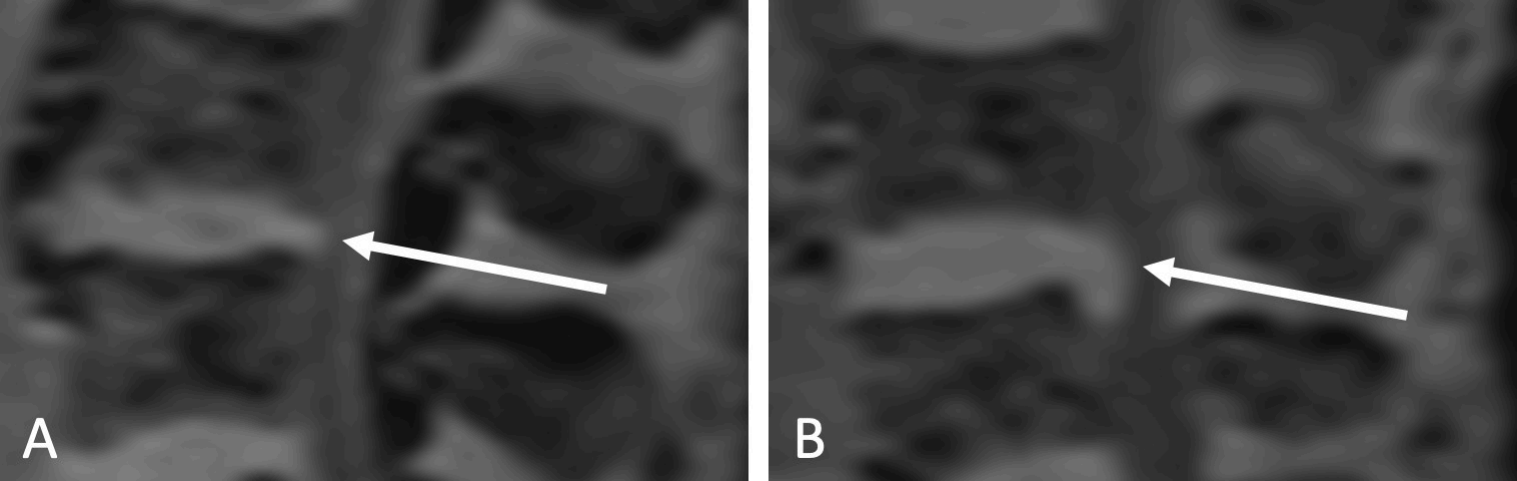
Image example of disc bulging (A) and disc protrusion (B) on L3/4 as indicated by the arrows (MRI sequence: VIBE Dixon Iso W).

Detectible tissue of the intervertebral discs in the spinal canal was assessed on a sagittal reconstructed dual-echo Dixon sequence and subclassified in either disc bulging, not exceeding the endplates of adjacent vertabrae, or disc protrusion, a perimeter of intervertebral discs exceeding the endplates of adjacent vertabrae (Fig 4) [32].

First, the frequency of disc bulging and protrusion were described on a per patient basis. Second, the number and location of disc bulging and protrusion were presented per patient.

### Image analysis

All datasets were independently assessed in a blinded and randomized fashion by a radiologist and an attending trauma surgeon with 5 and 6 years of experience in musculoskeletal imaging, respectively. In addition, to assess intra-reader agreement, 40 cases were randomly selected and re-evaluated in a blinded fashion by the primary reader (radiologist). Inter- and intra-reader agreement analysis of the spinopelvic parameters (PI, SS, PT, LL) revealed mean relative differences according to Bland-Altman analyses <5% for all parameters (inter-reader: -0.3%, 1.1%, -1.4%, 0.5%; intra-reader: -0.5%, 0.2%, -0.5%, -1.4%; respectively) and ICC values >0.95 (inter-reader: 0.99, 0.99, 0.98, 0.99; intra-reader: 0.99, 0.99, 0.98, 0.99; respectively). Inter- and intra-reader agreement analysis of the Pfirrmann Score was 85% (Kendall W = 0.93) and 87.5% (Kendall W = 0.92), respectively [7]. Inter- and intra-reader agreement analysis of disc bulging and disc protrusion showed an inter- and intra-reader agreement of 95% (Kappa = 0.84) for disc bulging and an inter- and intra-reader agreement of 100% (Kappa = 1.00) for disc protrusion.

### Statistical analysis

Characteristics of study participants including parameters of thoracolumbar disc degeneration and anatomical pelvis were summarized by means and standard deviations (SD) for continuous variables and counts and percentages for categorical variables. Comparisons between women and men were assessed by t-test or chi^2^-test, respectively. For descriptive purposes, the correlation of PI and LL was displayed graphically together with Pearson’s correlation coefficient.

Correlations of cardiovascular risk factors with the dependent outcome variables of MR-based spinopelvic parameters (PI, SS, PT, LL) were assessed by multivariable linear regression models providing β-coefficients with 95% confidence intervals (CI). The models included the risk factors age, sex, BMI, hypertension, total serum cholesterol, serum HDL-C, serum triglycerides, diabetes status, and physical activity. Associations of the spinopelvic parameters as independent exposure variables. The dependent outcome variable of MR-based summed continuous adapted Pfirrmann Scores (thoracic, lumbar and overall spine) was assessed by negative or zero inflated negative binomial regression models and incident rate ratios (IRR) with 95% CIs reported.

In order to estimate multivariable associations of the spinopelvic parameters with the outcome variables of individual spinal segment degeneration, disc protrusion, disc bulging, and back pain, logistic or ordered logistic regression models were applied and odds ratios with 95% CIs were calculated. All analyses were adjusted for the same cardiovascular risk factors mentioned above.

A p-value of <0.05 was considered to indicate statistical significance. In case of multiple testing, statistical significance was evaluated by p-value of <0.05 divided by the number of tests. All analyses were conducted using Stata 16.1 (Stata Corporation, College Station, TX, U.S.A.).

## Results

### General results

**Fig 5:**
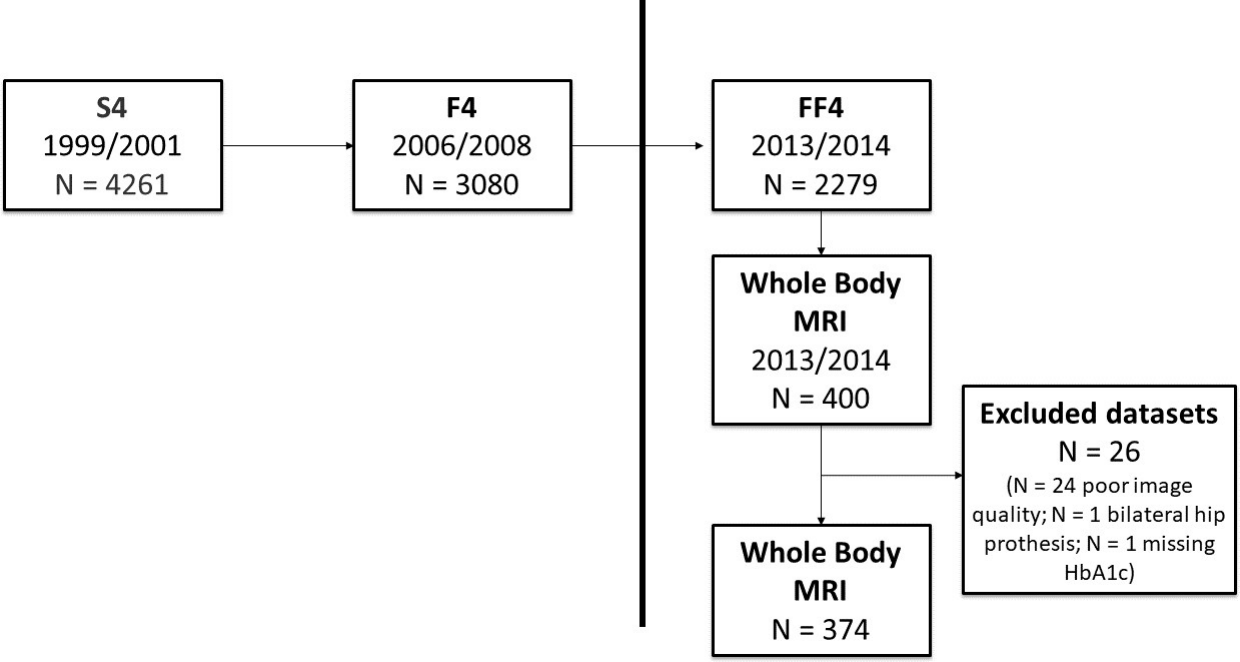
Flow chart of study participant selection. The vertical line represents the initial KORA-study with baseline examinations between 1999 and 2001 (S4 = Exam 1), first follow up between 2006 and 2008 (F4 = Exam 2) and second follow up between 2013 and 2014 (FF4 = Exam 3). Of the participants of the FF4 study, 400 were randomly selected to participate in the MRI sub-study (vertical line), and 26 subjects were excluded.

In total, 374 of the initial 400 participants were included in this study; 26 potential participants were excluded due to impaired image quality (n = 24), bilateral hip prostheses (n = 1) and missing serum HbA1c level (n = 1) (Fig 5).

The mean age of the cohort was 56.4 ± 9.2 years, 57.8% were male, mean BMI was 28.1kg/m^2^ ± 5.0, and back pain was present in 54.8% of all subjects.

Overall mean PI was 54.0° ± 11.1°, PT was 13.0° ± 5.8°, SS was 40.2° ± 8.8° and the LL was 36.2° ± 9.6°. SS was significantly greater in men (p<0.05), while lumbar lordosis was greater in women (p = 0.001). Detailed demographic and spinopelvic parameters are shown in Table 2.

**Table 2:** Descriptive demographic, spinopelvic and disc degeneration parameters of the study sample and according to gender.

Characteristics	All	Women	Men	
	N = 374	N = 158	N = 216	p
Age (years)	56.4 (±9.2)	56.5 (±9)	56.4 (±9.4)	0.91
BMI (kg/m^2^)	28.1 (±5.0)	27.5 (±5.5)	28.4 (±4.5)	0.09
Hypertension	130 (34.8%)	47 (29.8%)	83 (38.4%)	0.08
Total cholesterol (mg/dl)	218.1 (±36.7)	220.1 (±35.4)	216.7 (±37.6)	0.38
**HDL-C (mg/dl)**	**62.0 (±17.9)**	**70.6 (±17.9)**	**55.7 (±15.1)**	**<0.001**
LDL-C (mg/dl)	139.9 (±32.9)	137.7 (±32.7)	141.4 (±33)	0.29
**Triglyceride (mg/dl)**	**131.7 (±83.8)**	**102.0 (±45.9)**	**153.4 (±97.6)**	**<0.001**
HbA1c (%)	5.6 (±0.7)	5.6 (±0.6)	5.6 (±0.8)	0.81
**Diabetes status**				**0.002**
Prediabetes	91 (24.3%)	30 (19%)	61 (28.2%)	
Diabetes	52 (13.9%)	14 (8.9%)	38 (17.6%)	
Physical activity	224 (59.9%)	103 (65.2%)	121 (56%)	0.07
Back pain	205 (54.8%)	85 (53.8%)	120 (55.6%)	0.74
Mean adapted Pfirrmann Score	2.30 (±0.37)	2.33 (±0.40)	2.28 (±0.35)	0.19
Number of affected segments (discs)	4.3 (±5.6)	4.8 (±6)	3.9 (±5.3)	0.15
Disc Bulging	105 (28.1%)	42 (26.6%)	63 (29.2%)	0.58
Disc Protrusion	141 (37.7%)	57 (36.1%)	84 (38.9%)	0.58
Pelvic incidence (°)	54.0 (±11.1)	53.1 (±12.2)	54.6 (±10.2)	0.19
Pelvic tilt (°)	13.7 (±5.8)	13.7 (±6.5)	13.7 (±5.2)	0.93
**Sacral slope (°)**	**40.2 (±8.8)**	**39.2 (±9.2)**	**41.0 (±8.3)**	**0.045**
**Lumbar lordosis (°)**	**36.2 (±9.6)**	**38.1 (±9.9)**	**34.9 (±9.2)**	**0.001**

Data are given as mean ± standard deviation or number (percentage). Significant results are depicted in bold. P-values were calculated using t-testing or chi^2^-testing.

### Correlation between cardiovascular risk factors and spinopelvic parameters

Age was positively correlated with PT (an increase of 0.09 degrees per year of age) but not with PI, SS, or LL. There was a significant correlated between gender and sacral slope (β = 0.47; 95%CI 0.27 to 0.81, p<0.05) and lumbar lordosis (β = -3.33; 95%CI -5.5 to -1.15, p<0.01), with men having a greater sacral slope but less lumbar lordosis compared to women. Neither BMI, hypertension, serum cholesterol level, serum lipid level, nor degree of physical activity were correlated with any spinopelvic parameters. However, a negative significant correlation between diabetes mellitus and the sacral slope (β = -4.19; 95%CI -7.31 to 1.06, p<0.01) was observed (Table 3).

**Table 3:** Multivariable correlation of risk factors with spinopelvic parameters.

Risk factors	Pelvic Incidence		Pelvic Tilt		Sacral Slope		Lumbar Lordosis	
	β (95% CI)	*p*	β (95% CI)	*p*	β (95% CI)	*p*	β (95% CI)	*p*
Age (years)	-0.01 (-0.15; 0.13)	0.92	0.09 (0.02; 0.17)	0.011	-0.10 (-0.21; 0.00)	0.061	0.05 (-0.06; 0.17)	0.37
Men	1.80 (-0.77; 4.37)	0.17	0.06 (-1.28; 1.39)	0.94	**2.05 (0.07; 4.04)**	**0.043**	**-3.33 (-5.5; -1.15)**	**0.003**
BMI (kg/m^2^)	0.13 (-0.14; 0.4)	0.35	0.01 (-0.13; 0.15)	0.88	0.11 (-0.10; 0.32)	0.292	0.19 (-0.04; 0.42)	0.11
Hypertension	0.70 (-2.08; 3.47)	0.62	-0.47 (-1.91; 0.96)	0.52	1.29 (-0.85; 3.44)	0.237	0.92 (-1.43; 3.26)	0.44
Total cholesterol (mg/dl)	0.01 (-0.03; 0.04)	0.63	0.00 (-0.02; 0.01)	0.66	0.01 (-0.02; 0.04)	0.373	0.01 (-0.02; 0.04)	0.46
HDL-C (mg/dl)	0.03 (-0.06; 0.12)	0.48	0.01 (-0.03; 0.06)	0.64	0.02 (-0.05; 0.09)	0.526	0.03 (-0.05; 0.10)	0.48
Triglyceride (mg/dl)	0.01 (-0.01; 0.03)	0.31	0.00 (-0.01; 0.01)	0.67	0.01 (-0.01; 0.02)	0.290	0.01 (-0.01; 0.02)	0.44
Diabetes status								
Prediabetes	-1.69 (-4.74; 1.36)	0.28	-0.47 (-2.05; 1.11)	0.56	-1.38 (-3.74; 0.98)	0.250	0.75 (-1.83; 3.33)	0.57
Diabetes	-2.70 (-6.75; 1.34)	0.19	1.14 (-0.96; 3.24)	0.29	**-4.19 (-7.31; -1.06)**	**0.009**	-0.22 (-3.64; 3.20)	0.90
Physical activity	0.87 (-1.57; 3.31)	0.48	0.95 (-0.32; 2.22)	0.14	-0.02 (-1.91; 1.87)	0.984	0.88 (-1.19; 2.95)	0.40

β-coefficients are from multivariable linear regression models. Significant values are in bold. CI: Confidence interval.

### Correlation of spinopelvic parameters with disc degeneration as assessed by adapted Pfirrmann-Score

No significant correlation was observed between PI, PT, SS, and LL and degenerative changes of the thoracic, lumbar, or entire thoracolumbar spine (Table 4a and 4b).

**Table 4:** a. Multivariable correlation of spinopelvic parameters with number of affected segments (discs). **b.** Multivariable correlation of spinopelvic parameters with mean overall Pfirrmann Score.

Risk factors	Thoracic adapted Pfirrmann Score Th1-12*		Lumbar adapted Pfirrmann Score L1-5		Overall adapted Pfirrmann Score (Th1-12 and L1-5)	
	IRR (95% CI)	*p*	IRR (95% CI)	*p*	IRR (95% CI)	*p*
Pelvic incidence (PI)	0.99 (0.98; 1.01)	0.48	0.99 (0.98; 1.00)	0.18	1.00 (0.98; 1.01)	0.47
Pelvic tilt (PT)	0.99 (0.96; 1.03)	0.70	1.00 (0.98; 1.02)	0.86	1.00 (0.98; 1.02)	0.98
Sacral slope (SS)	0.99 (0.97; 1.01)	0.36	0.99 (0.98; 1.00)	0.06	0.99 (0.98; 1.01)	0.25
Lumbar lordosis (LL)	1.00 (0.98; 1.02)	0.85	0.99 (0.98; 1.00)	0.12	1.00 (0.98; 1.01)	0.71
Pelvic incidence (PI)	1.00 (0.99; 1.01)	0.95	1.00 (0.99; 1.00)	0.61	1.00 (0.99; 1.01)	0.84
Pelvic tilt (PT)	1.00 (0.99; 1.01)	0.88	1.00 (0.99; 1.01)	0.86	1.00 (0.99; 1.01)	0.96
Sacral slope (SS)	1.00 (0.99; 1.01)	0.99	1.00 (0.99; 1.00)	0.44	1.00 (0.99; 1.01)	0.80
Lumbar lordosis (LL)	1.00 (0.99; 1.01)	0.95	1.00 (0.99; 1.01)	0.59	1.00 (0.99; 1.01)	0.90

Incident rate ratios (IRR) are from (*zero inflated) negative binomial regression models adjusted for age, sex, body-mass index, hypertension, total serum cholesterol, serum HDL-C, serum triglycerides, diabetes status, and physical activity.

### Correlation between spinopelvic parameters and disc protrusion and disc bulging

Pelvic incidence (p<0.01), sacral slope (p<0.05) and lumbar lordosis (p<0.01) were inversely correlated with disc bulging. A more frequent occurrence of disc bulging was seen in participants with lower pelvic incidence, lower sacral slope and lower lumbar lordosis, indicating that less lordosis of the spine is associated with disc bulging. Pelvic tilt was borderline non-significantly correlated with disc bulging. There was no association between spinopelvic parameters and disc protrusion (Table 5A–5D).

**Table 5:** a. Multivariable correlation of spinopelvic parameters with disc protrusion and disc bulging independent of the number of disc herniation per patient. **b.** Multivariable correlation of spinopelvic parameters with disc protrusion and disc bulging depending on the number of disc herniation per patient. **c.** Multivariable correlation of spinopelvic parameters with disc protrusion depending on the location (thoracic: Left; lumbar: Right). **d.** Multivariable correlation of spinopelvic parameters with disc bulging depending on the location (thoracic: Left; lumbar: Right).

Risk factors	Disc protrusion		Disc bulging	
	OR (95% CI)	p	OR (95% CI)	p
Pelvic incidence (PI)	1.00 (0.98; 1.02)	0.69	**0.97 (0.95; 0.99)**	**0.009**
Pelvic tilt (PT)	1.00 (0.97; 1.04)	0.88	0.96 (0.92; 1.00)	0.05
Sacral slope (SS)	0.99 (0.97; 1.02)	0.53	**0.97 (0.94; 1.00)**	**0.035**
Lumbar lordosis (LL)	1.01 (0.98; 1.03)	0.55	**0.96 (0.93; 0.98)**	**0.002**
**Risk factors**	number **Disc protrusion**		number **Disc bulging**	
	OR (95% CI)	p	OR (95% CI)	p
Pelvic incidence (PI)	1.00 (0.98; 1.02)	0.84	0.97 (0.95; 0.99)	**0.007**
Pelvic tilt (PT)	1.01 (0.97; 1.05)	0.65	0.96 (0.92; 1.00)	0.06
Sacral slope (SS)	0.99 (0.97; 1.02)	0.57	0.97 (0.95; 1.00)	**0.031**
Lumbar lordosis (LL)	1.01 (0.99; 1.03)	0.50	0.96 (0.93; 0.98)	**0.001**
	**Disc protrusion**			
**Risk factors**	location (**Th1-12)**		location (**L1-5)**	
	RRR (95% CI)	p	RRR (95% CI)	p
Pelvic incidence (PI)	0.99 (0.94; 1.05)	0.73	1.00 (0.98; 1.02)	0.71
Pelvic tilt (PT)	0.99 (0.90; 1.09)	0.82	1.00 (0.96; 1.04)	0.88
Sacral slope (SS)	0.99 (0.92; 1.06)	0.79	0.99 (0.97; 1.02)	0.55
Lumbar lordosis (LL)	1.01 (0.94; 1.08)	0.874	1.01 (0.98; 1.03)	0.57
	**Disc bulging**			
**Risk factors**	location (**Th1-12)**		location (**L1-5)**	
	RRR (95% CI)	p	RRR (95% CI)	p
Pelvic incidence (PI)	1.00 (0.9; 1.11)	1.0	0.97 (0.95; 0.99)	**0.008**
Pelvic tilt (PT)	0.96 (0.77; 1.19)	0.69	0.96 (0.92; 1.00)	0.06
Sacral slope (SS)	1.02 (0.89; 1.17)	0.79	0.97 (0.94; 1.00)	**0.029**
Lumbar lordosis (LL)	1.07 (0.95; 1.19)	0.26	0.95 (0.93; 0.98)	**0.001**

Odds ratios are from logistic regression models adjusted for age, sex, body-mass index, hypertension, total cholesterol, HDL-C, triglycerides, diabetes status, physical activity.

Relative risk ratios are from multinomial logistic regression models adjusted for age, sex, body-mass index, hypertension, total cholesterol, HDL-C, triglycerides, diabetes status, physical activity.

Odds ratios are from ordered logistic regression models adjusted for age, sex, body-mass index, hypertension, total cholesterol, HDL-C, triglycerides, diabetes status, physical activity.

Relative risk ratios are from multinomial logistic regression models adjusted for age, sex, body-mass index, hypertension, total cholesterol, HDL-C, triglycerides, diabetes status, physical activity.

Smaller spinopelvic parameters (PI, SS and LL) where significantly correlated with an increased frequency of disc bulging (Table 5B) as well as a local clustering in the lumbar, but not the thoracic spine (Table 5D).

### Correlation between spinopelvic parameters and back pain

No significant correlation between pelvic incidence, pelvic tilt, sacral slope, or lumbar lordosis and back pain was observed (Table 6). In total, 205 participants (54.8%) complained of back pain (little: 32.9%; medium: 17.4%; strong: 3.7%; very strong: 0.8%).

**Table 6:** Multivariable correlation of spinopelvic parameters with back pain.

Risk factors	Back pain*^#^	
	OR (95% CI)	p
Pelvic incidence (PI)	1.00 (0.98; 1.02)	0.93
Pelvic tilt (PT)	1.01 (0.97; 1.04)	0.70
Sacral slope (SS)	1.00 (0.98; 1.02)	0.83
Lumbar lordosis (LL)	0.99 (0.97; 1.01)	0.60

Odds ratios are from (^#^ordered) logistic regression models adjusted for age, sex, body-mass index, hypertension, total serum cholesterol, serum HDL-C, serum triglycerides, diabetes status, degree of physical activity.

*(Not at all, little, medium, strong, very strong).

**Fig 6:**
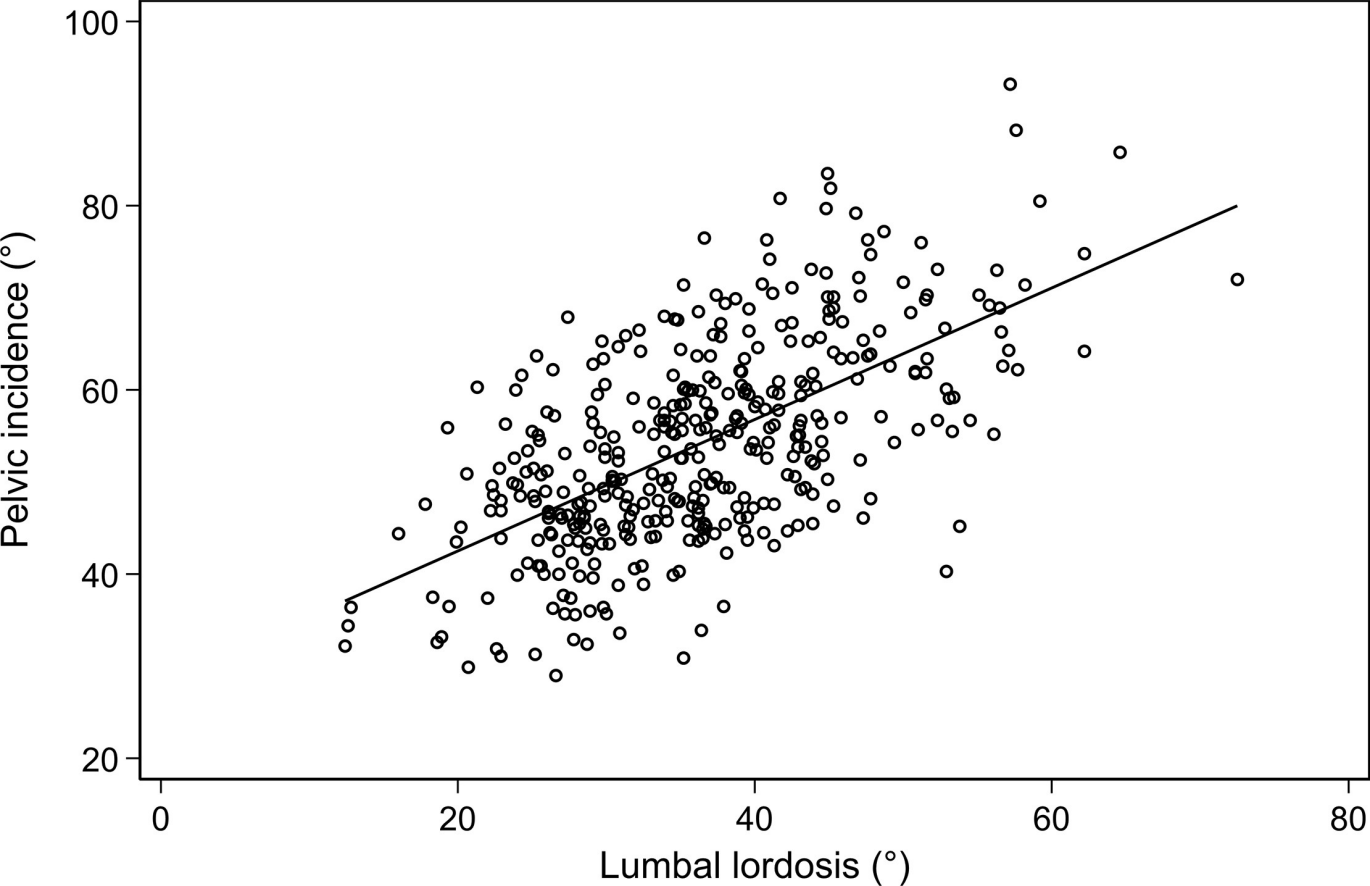
Correlation of pelvic incidence and lumbar lordosis, r = 0.614, p<0.001.

There was a positive correlation (r = 0.614, p<0.001) between pelvic incidence and lumbar lordosis in the cohort (Fig 6).

## Discussion

In this study, none of the spinopelvic parameters (PI, PT, SS or LL), measured in supine position, influenced disc degeneration of the thoracolumbar spine significantly. In addition, no correlation between back pain and the spinopelvic parameters could be found. A significant correlation between pelvic tilt and age could be demonstrated, with an increase in degrees per age-year. Gender was significantly correlated with sacral slope and lumbar lordosis, with men having a greater sacral slope but a smaller lumbar lordosis compared to females. PI, SS and LL were significantly correlated with disc bulging, whereby smaller angles showed an increase in the frequency of disc bulging, as well as a local clustering in the lumbar, but not the thoracic spine.

Recently, experts have speculated that spinopelvic parameters affect axial loading, which in turn leads to degenerative disc disease and low back pain if not properly aligned, with PI having the leading role by determining the thoracolumbar curve [14,18]. The fact, that the pelvic parameters influence the posture of the spine is evident in the literature. Various authors have already shown, that a small PI angle leads to a decrease in SS. This in turn results in a flattening of the LL and vice versa [20,22,23], which is consistent with the results of this study.

Nevertheless, the present study showed no correlation with supine spinopelvic parameters and disc degeneration or back pain. Consequently, it may not be likely that the supposed relationship between spinal alignment and axial loading as defined by spinopelvic parameters are involved in the underlying pathology of disc degeneration. In addition, Roussouley already described four different norm variants of the spine, none of which was to be considered pathological, despite the different expression of the curvature and slope [19,24].

However, literature is divided on this point. A small PI, of which a lower SS is a consequence, leads to a flattening of the LL, thus resulting in a less curved spine. Consequently, the force of gravity increases the pressure on the intervertebral discs, causing degeneration of the intervertebral discs [14,23]. The capacity to absorb axial impact loads decreases, which further promotes the process of degeneration and can even lead to disc herniation. Vice versa, degenerative disc changes, with a reduction of the disc height, can lead to a decrease of the LL [23]. Habibi et al. likewise found that patients with degenerative disc alterations show a more upright and therefore less curved spine [33].

In this study disc bulging was significantly more often present in patients with smaller PI, SS and LL. Especially small PI, SS and LL values were found to significantly cluster disc bulging in the lumbar but not the thoracic spine. These findings were concordant with those from Yang et al. [23] and Barrey et al. [14], with a normal or lowered PI in patients with lumbar disc herniation.

It is assumed, especially in physiotherapy, that back pain can be caused by a pronounced lumbar lordosis. The results of this study did not prove any correlation between the supine spinopelvic parameters and back pain. This is opposed by the findings of Yang et al., showing that symptomatic patients had smaller SS and LL, but higher PT values [23]. The overview by Chun et al. also showed that back pain is correlated with a small LL, whereby degenerative concomitant diseases of the spine (disc degeneration, spondylolistheses or herniation of the intervertebral discs) contribute to a variance in the different trial results [34]. Besides, Barrey et al. showed that besides degenerative structural changes in spinal alignment, pain-related postural changes must also be considered (e.g. bending posture of the spine to relieve pain in spinal canal stenosis) [14].

A significant association with age was only shown for PT, with a steady increase of 0.09 degrees per year of life. Consistent with our findings, the results of Asai et al. prove that PT increases with age [35]. However, the increase remains at 0.1 degrees over 10 years. Furthermore, already little changes in positioning during MRI can cause an increase (retroversion of the pelvis) or decrease (anteversion of the pelvis) of PT. Therefore, we do not consider this to be relevant [28]. Contrary to expectations, this study did not demonstrate a significant association between age and PI. This might be due to the fact that in the present cohort only adult individuals were included and thus the developmental change in PI was not registered [14,19,20]. In addition, patients showed a decrease in LL with increasing age, with the decrease in LL being more prominent in women [35]. Decrease in lumbar lordosis with age can lead to a kinematic alteration and thus to an accelerated degeneration of the spine due to a change in axial load [33].

In this study cohort, SS was substantially greater in men, whereas women exhibited a significant higher LL. These findings are similar to those of the Wakayama Spine study, as PI, PT and LL differed slightly between sexes [35].

Still, the literature remains controversial, Vialle and colleagues showed larger SS in women than in men [36], while Jackson et al. did not find any significant difference in LL and thoracic kyphosis between the sexes [37].

The results of this study did not demonstrate any significant correlation between BMI and supine PI, PT, SS and LL. However, the literature is divided on this point. Boulay et al. showed a strong correlation between BMI and PI, LL and SS, substantiating the association to mechanical restrictions in obese patients [22].

In contrast, other studies showed that there was no significant correlation between obesity and PI, PT, LL [38,39] and SS [39]. However, Romero-Vagas et al. admitted that, despite the lack of significance, a trend towards higher values could be observed in overweight patients and drew the conclusion that obesity exerts greater shear forces on the lumbosacral junction leading to a considerably more fragile lumbosacral connection [39].

This study has limitations. First, the spinopelvic parameters were acquired on MRI datasets with participants in supine position. Andreasen et al. demonstrated that with appropriate supine positioning it is possible to imitate an upright position and thus generate a similar lumbar lordosis [40]. Chevillotte et al. proved that PI had a similar value in standing, seating and lying position [41]. In addition, PT and SS were slightly different but still comparable in standing and supine positions, whereas clearer deviations were evident in seated positions [41]. To ensure good comparability of results, all patients were positioned for MRI using a standardized protocol.

Second, MRI was only performed once at FF4 and therefore this is a cross-sectional study. A long-term analysis on the progression of disc degeneration as a function of spinopelvic parameters is not possible. Further long-term follow-ups may provide additional information. Third, the adapted Pfirrmann score assesses the intervertebral discs on visual parameters; not including the adjacent bony structures. Since there are no metric measurements performed to classify the Pfirrmann grades, correlation between readers may be impacted by the clinical experience of the readers.

In conclusion, spinopelvic parameters, measured in supine position, are significantly associated with disc bulging alone; there is no significant correlation between supine spinopelvic parameters and disc degeneration, back pain or cardiovascular risk factors.
